# Children Use Wealth Cues to Evaluate Others

**DOI:** 10.1371/journal.pone.0149360

**Published:** 2016-03-02

**Authors:** Kristin Shutts, Elizabeth L. Brey, Leah A. Dornbusch, Nina Slywotzky, Kristina R. Olson

**Affiliations:** 1 University of Wisconsin-Madison, Department of Psychology, Madison, Wisconsin, United States of America; 2 Yale University, Department of Psychology, New Haven, Connecticut, United States of America; 3 University of Washington, Department of Psychology. Seattle, Washington, United States of America; University of Tuebingen Medical School, GERMANY

## Abstract

Wealth differences between individuals are ubiquitous in modern society, and often serve as the basis for biased social evaluations among adults. The present research probed whether children use cues that are commonly associated with wealth differences in society to guide their consideration of others. In Study 1, 4–5-year-old participants from diverse racial backgrounds expressed preferences for children who were paired with high-wealth cues; White children in Study 1 also matched high-wealth stimuli with White faces. Study 2 conceptually replicated the preference effect from Study 1, and showed that young children (4–6 years) also use material wealth indicators to guide their inferences about people’s relative standing in other domains (i.e., competence and popularity). Study 3 revealed that children (5–9 years) use a broad range of wealth cues to guide their evaluations of, and actions toward, unfamiliar people. Further, biased responses were not attenuated among children whose families were lower in socioeconomic status. Often overlooked by those who study children’s attitudes and stereotypes, social class markers appear to influence evaluations, inferences, and behavior early in development.

## Introduction

In January 2015, Oxfam released a report announcing that the 80 richest individuals in the world now hold more wealth than the poorest 3.5 *billion* people on earth [1]. Within the United States, economic inequality is high, with households in the top income quintile earning over $100,000 per year and households in the bottom income quintile earning less than a fifth of that [2]. Such disparities not only fuel heated debates about economic policy and drive social movements, but also ignite clashes between citizens who are more or less well-off. Terms noting social divides are ubiquitous (e.g., “class warfare”; “the 99%”), as are phrases derogating people from different socioeconomic backgrounds (e.g., “welfare queen”). Thus, today’s children are born into a world where economic inequality is prominent and where the gap between rich and poor is increasing. The current paper asks what role, if any, social class distinctions play in guiding children’s evaluations of, inferences about, and actions toward, other people.

Economic issues are complex and are also most directly relevant to those who can vote, earn income, and hold wealth. It would therefore be reasonable to think that only adults are concerned with socioeconomic status (SES). Indeed, classic interview studies conducted by Leahy in the 1980s with children between 5 and 18 years suggested that young children have difficulty thinking about economic concepts: Most participants could not generate sensible explanations for wealth differences prior to 11 years of age ([3–5]; see also [6–8]). Further, when asked to describe “rich” and “poor” people, young children (unlike older children and adolescents) rarely mentioned psychological attributes; rather, they often said, “I don’t know” or referenced money and possessions ([3]; see also [9]).

While interview studies suggest that wealth is not a particularly salient or meaningful social distinction until late in development, other lines of research point to a different conclusion. Preschool-age children can classify photographs of unfamiliar people as “rich” or “poor” [6]. Additionally, using methods simpler than those employed by Leahy, studies have shown that kindergarteners think that poverty is heritable and stable across the lifespan [10]. Further, children as young as 5 years of age think that people with similar levels of wealth are likely to have other properties in common (e.g., activity preferences; [10–11]). Taken together, these studies indicate that children can use wealth information to classify and reason about other people early in childhood. However, this research does address whether young children use wealth information to guide their evaluations of, or behavior toward, others.

There is ample evidence that by adulthood, people are more favorable toward those who are higher in SES (for reviews, see [12–13]). A small body of literature suggests that some of the class-based attitudes and stereotypes held by adults are also evident in elementary school-age children: A recent study of Black and multi-racial South African children’s social class attitudes found that 6- to 12-year-old participants were more positively disposed toward children who were shown to be wealthy over children who were shown to be poor [14]. In a study of U.S. children’s stereotypes, Baldus & Tribe [15] showed that elementary school-age participants thought that a child from a poor family would be more likely than a child from a wealthier family to get into a fight, perform poorly on a reading test, tell lies to his family, and have few friends. Additionally, in more recent research, Sigelman [7] found that 1^st^, 5^th^, and 9^th^ grade U.S. students who heard stories about a man described as either “rich” or “poor” subsequently rated the rich man more positively than the poor man on traits that referred to intelligence, motivation, and responsible behavior. In contrast to the study’s hypotheses, 1^st^ grade participants rated the poor man more favorably than the rich man on traits that referred to social attractiveness (e.g., “nice”), while 5^th^ and 9^th^ grade participants rated the two men equally.

Whether younger children use wealth information to evaluate individuals remains unexplored, however. Thus, the main aim for the present research was to probe young children’s evaluations of those who differ in wealth. We employed a variety of measures across three studies with children who varied by age and social background. In every study, we avoided using the terms “rich” or “poor” to describe target stimuli, reasoning that children often encounter people in the absence of such labels—on television, at the park, and in school. Further, previous research shows that providing labels can heighten children’s attention to social distinctions and invite them to make inferences they would not otherwise generate [16–17]. Labeling could thereby inflate or even potentiate social class bias in children.

Our method for conveying wealth information in all three studies was to present pictures of unfamiliar individuals paired with cues that are commonly associated with higher or lower wealth (e.g., a large house with a manicured lawn vs. a small, simple house). For ease of exposition in the manuscript, we sometimes refer to the “high-wealth individual” or the “low-wealth individual” or to children’s “preference for the wealthier person,” but of course stimuli cannot be wealthy or poor. We never labeled the stimuli as such for children, and we cannot know from the work presented here how similar children’s interpretation of our stimuli is to adults’ interpretation. We return to these important issues in the General Discussion.

## Study 1

Study 1 investigated social class bias in the youngest children tested to date—preschoolers. To do this, we adapted methods commonly used to assess preschool-age children’s social category-based preferences: In studies of young children’s gender and race attitudes, participants are often presented with pairs of photographs that differ along the dimension of interest (e.g., one Black and one White individual, or one boy and one girl), and are asked to point to the person they like or to the person they would like to befriend (see, for example [18–19]). Accordingly, for the “wealth preference task” in Study 1, 4–5-year-old participants viewed photographs of unfamiliar children who appeared to differ in wealth and were asked to indicate whom they liked. We used material possessions (houses and personal effects) to convey wealth differences, as previous research has shown that young children are sensitive to such visual markers [6,20–21]. Such depictions are also ecologically valid, as wealthy families are, in fact, more likely than poor families to own material goods that are new and branded [22–23].

Although the primary goal of Study 1 was to test whether young children show preferences for people based on cues to wealth, we also included another measure of children’s consideration of wealth information: The “wealth/race matching task” assessed whether, in addition to showing wealth-based preferences, children link wealth differences to another distinction that is present in the real world and that serves to guide young children’s attitudes and stereotypes: namely, race. On every trial, participants saw two families (one Black and one White) and two kinds of wealth stimuli (low- and high-wealth), and were asked to match families with their houses or their things. Elementary school-age children in the United States and South Africa think that White people are likely to be higher in SES than Black or multiracial people [14,24–27]. In Study 1, we asked whether younger children similarly link race and social class.

### Method

#### Participants

The participants were 102 children (38 boys, 64 girls) ages 4–5 years (*M* age = 57 months; range = 48–71). The sample was comprised of 47 White children, 31 Black children, and 24 children from a wide array of other racial and ethnic groups. Approximately one quarter of participants (15 Black children, 8 multiracial/other) were tested in schools in the Midwestern region of the U.S., while remaining participants (47 White children, 16 Black children, 16 multiracial/other) were tested in schools and afterschool programs in the Northeastern region of the U.S.

We were unable to obtain family SES information for individual participants in the study. However, 52 participants (9 White, 30 Black, 13 from other racial/ethnic groups) were tested in locations serving predominantly low-SES families (e.g., Head Start programs); 35 participants (29 White, 1 Black, 5 from other racial/ethnic groups) were tested in locations serving predominantly middle-SES families; and 15 participants (9 White, 6 from other racial/ethnic groups) were tested in locations serving predominantly high-SES families (e.g., expensive private preschools).

The study was approved by the University of Wisconsin-Madison Social and Behavioral Sciences IRB [Protocol #2012–0764] and the Yale University IRB [Protocol #0807004095]). The legal guardians of all participants provided written informed consent, and children provided verbal assent.

#### Materials

For both the wealth preference task and the wealth/race matching task, participants viewed photographed faces of unfamiliar Black and White people. Faces within a trial were matched according to adult judgments of attractiveness, expression (all bore positive facial expressions), and age. Photographs were never repeated across tasks or across trials within a task. Both tasks also presented photographs of real houses and objects in order to depict differences in wealth. All photographs of people, houses, and objects were obtained through Internet searches.

Objects for the wealth preference task were backpacks, shoes, sports equipment, and jeans, while objects for the wealth/race matching task were cars, backyard play-sets, electronics, and vacation destinations. “High-wealth” objects looked new, while “low-wealth” objects looked used (examples: a brand new luxury car vs. a noticeably used older car; new designer blue jeans vs. older generic jeans). Object stimuli were the same for children in the Midwest and Northeast, but house photographs differed in accordance with what was typical for higher- and lower-income residences in each geographical region (as observed in Internet searches for houses on the market in both regions): For participants tested in the Northeast, “high-wealth” and “low-wealth” houses were similar in size, but the former were well maintained (e.g., fresh paint) and the latter showed visible signs of wear-and-tear (e.g., crooked porch railings). For participants tested in the Midwest, “high-wealth” houses were large and had manicured yards, while “low-wealth” houses were small and simple. Adult judgments were obtained for each of the house and object pairs used in Study 1; these raters (*N* = 8) thought that the “high-wealth” stimulus was more likely to belong to a person or family with a higher level of wealth 99% of the time.

#### Procedure

Participants were tested individually in a quiet room at their school or in the lab. A Black female experimenter tested Black participants, while a White female experimenter tested all remaining participants. The experimenter never provided feedback on children’s choices during the tasks, and never used labels (e.g., “Black”, “poor”) to refer to stimuli. The wealth/race matching task always preceded the wealth preference task. The tasks were ordered in this manner because of concerns that seeing Black and White individuals associated with wealth information in the preference task might interfere with participants’ inferences about links between wealth and race in the matching task.

[Participants also completed measures designed to assess their gender- and race-based social preferences. These measures were completed before the tasks described here. We have opted not to describe the tasks in detail, as they are not relevant to the focus of the present paper and merely replicate extant findings. However, given increasing concerns in the field concerning replication, as well as the recommendation to report all measures [28], we note the results for the measures here: As in past research, participants preferred same-gender over other-gender children (*p* < .001), and this effect was stronger among girls (*p* < .001). Additionally, participants preferred White over Black children (*p* < .001); this tendency was somewhat more pronounced among White than among Black children (*p* = .092).]

**Fig 1:**
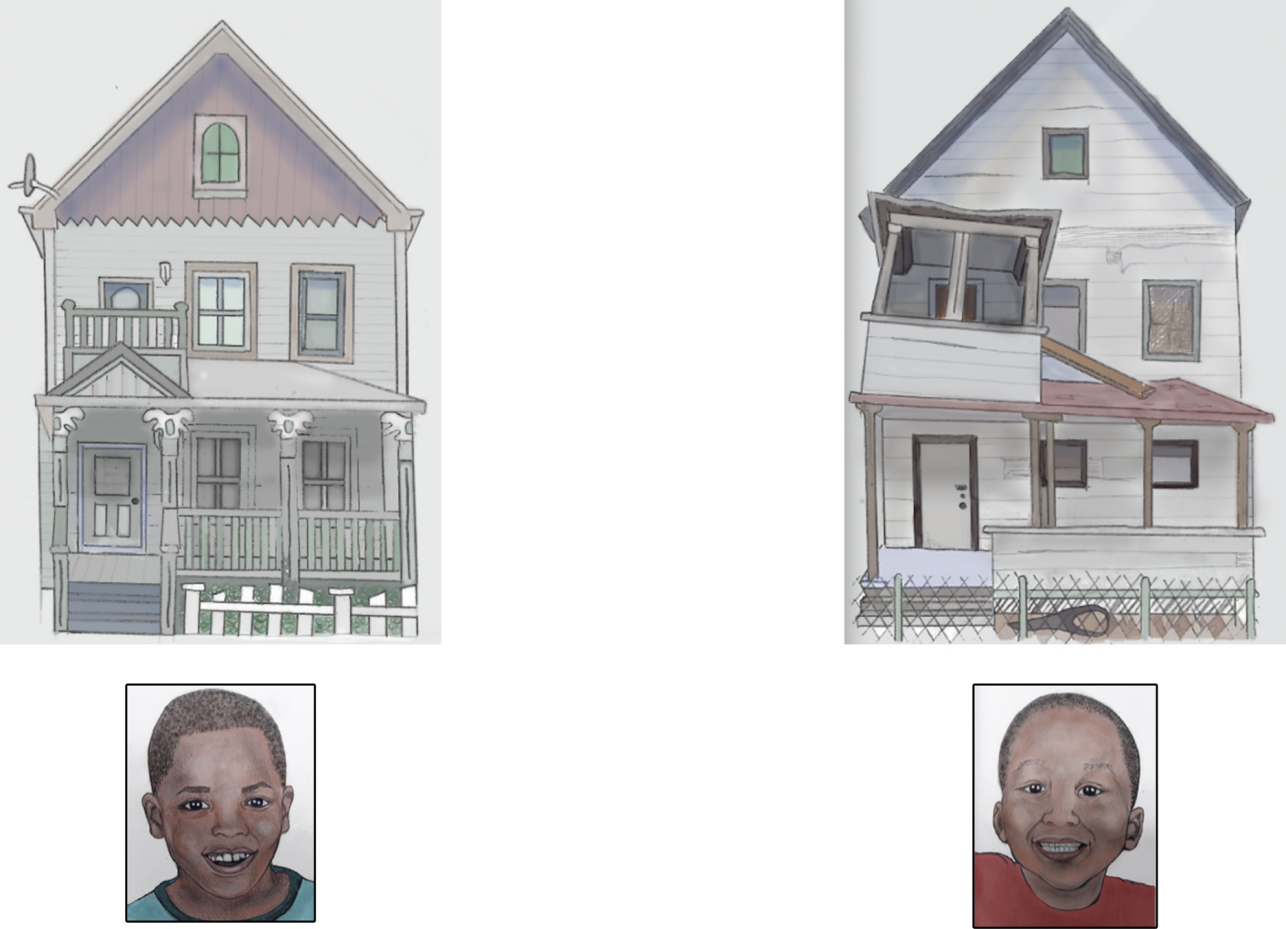
Example house trial from the wealth preference task in Study 1. The child on the left is shown with a house meant to connote high wealth, while the child on the right is shown with a house meant to connote lower wealth. Because we do not have permission to publish the images of houses, objects, and faces that were used in the task, the figure shows an artist’s rendition of a display from the task. Participants in the study saw color photographs of real houses, objects, and faces.

Wealth preference task: On every trial, participants saw photographs of two children who matched one another in race and gender, arranged horizontally. A low-wealth house or object appeared directly above one child, while a higher-wealth house or object appeared above the other child (Fig 1). On each trial, the experimenter pointed to the display and said, “See this kid and see this kid? This kid lives in this house (or wears/uses this), and this kid lives in this house (or wears/uses this).” Participants were then asked to point to the child they liked more.

There were 8 unique trials in the wealth preference task. Four trials included a pair of White children (two female pairs, two male pairs), and four included a pair of Black children (two female pairs, two male pairs). These different pair types were interspersed throughout the task. Half of the trials of each race and gender presented two houses to depict wealth differences, and half presented two objects. The order of trials—including whether a house or object trial came first, whether children saw a Black or White pair first, and whether children saw a female or male pair first—was counterbalanced across children. Pairings of particular houses/objects with particular face pairs, as well as pairings of high-wealth and low-wealth objects with particular faces within a pair, were also counterbalanced across children. The lateral positions of high- and low-wealth targets were counterbalanced within and across participants.

Wealth/race matching task: At the outset of each trial, the experimenter presented participants with one low-wealth house or object alongside one high-wealth house or object. The experimenter pointed to each house/object and said, “See this house (or thing)? See this house (or thing)?” Then the experimenter showed participants pictures of one White family and one Black family. The families appeared below the houses/objects, and were arranged vertically so that each family was equidistant from each of the houses/objects. Participants were asked to put the families in their houses (on trials showing two houses) or to put the families with their things (on object trials).

There were 8 unique trials in the wealth/race matching task: Four trials presented houses to depict wealth differences, and four presented objects. Half of each type of trial presented the White family above the Black family and half presented the Black family above the White family. The lateral positions of the high- and low-wealth houses/objects were counterbalanced within and across participants. Trial order and pairings of particular family pairs with particular house/object pairs were counterbalanced across participants. All families had at least one parent and one child, but the composition of the families varied across trials in the task (i.e., some families had two parents and one child, some had two parents and two children, and some one parent and two children). Within each trial, however, Black and White family compositions were matched.

#### Scoring and analyses

For the wealth preference task, selecting the child depicted with the high-wealth item was scored as “1,” while selecting the child depicted with the low-wealth item was scored as “0.” For the wealth/race matching task, trials where participants matched the White family with the high-wealth item (and therefore matched the Black family with the low-wealth item) were scored as “1”, while trials where participants matched the Black family with the high-wealth item (and therefore matched the White family with the low-wealth item) were scored as “0”. Total possible scores for each task ranged from “0” to “8.” We also created sub-scores for each task to assess the impact of depicting wealth information with objects vs. with houses (range = “0” to “4”). Analyses considering participant race focused only on Black and White participants because there were not enough participants from any other particular racial or ethnic group to include.

### Results

#### Wealth preference task

Three participants did not want to complete the preference task. Remaining participants (*N* = 99) tended to prefer children paired with high-wealth cues over children paired with low-wealth cues (Chance = 4; *M* = 5.10, *SD* = 1.54, *t*(98) = 7.11, *p* < .001, *d* = .71). Scores were higher when wealth information was conveyed with objects compared to houses (*t*(98) = 3.04, *p* = .003, *d*_*z*_ = .31). Nevertheless, performance on both kinds of trials was above chance (Chance = 2; object trials: *M* = 2.76, *SD* = 1.06, *t*(98) = 7.11, *p* < .001, *d* = .71; house trials: *M* = 2.34, *SD* = .99, *t*(98) = 3.45, *p* = .001, *d* = .35). Black and White participants’ responses did not differ (*p* = .939, *d* = .02), and a one-way ANOVA showed no effect of school SES (low vs. middle vs. high) on participants’ responses (*p* = .622, η_p_^2^ = .01).

#### Wealth/race matching task

One participant did not want to complete the task. Remaining participants (*N* = 101) showed a significant tendency to match White families with high-wealth items (Chance = 4; *M* = 5.16, *SD* = 1.85, *t*(100) = 6.30, *p* < .001, *d* = .63), and performance did not depend on whether trials featured objects or houses (*p* = .937, *d*_*z*_ = .01). White participants’ scores were higher than those of Black participants^2^, indicating that they more strongly associated White families with wealth than did Black participants (*M* = 5.65, *SD* = 1.69 and *M* = 4.39, *SD* = 1.78, respectively; *t*(75) = 3.15, *p* = .002, *d* = .74). According to one-sample *t* tests, only White participants’ performance on the wealth/race matching task differed from chance (White participants: *t*(45) = 6.63, *p* < .001, *d* = .98; Black participants: *t*(30) = 1.21, *p* = .236, *d* = .22).

A one-way ANOVA showed an effect of school SES (*F*(2,98) = 7.75, *p* < .001, η_p_^2^ = .14). LSD post-hoc tests indicated that the scores of participants tested at low-SES schools were lower than the scores of those attending both middle-SES and high-SES schools (*p* = .003 and *p =* .001, respectively); responses from participants at middle- and high-SES schools did not differ (*p* = .333). According to one-sample *t* tests, scores of participants at low-, middle- and high-SES schools exceeded chance (*M*_Low_ = 4.52, *SD* = 1.78, *t*(51) = 2.11, *p* = .040, *d* = .29; *M*_Middle_ = 5.68, *SD* = 1.77, *t*(33) = 5.52, *p* < .001, *d* = .95; *M*_High_ = 6.20, *SD* = 1.47, *t*(14) = 5.78, *p* < .001, *d* = 1.49).

### Discussion

Participants in Study 1 preferred children who were presented with high-wealth cues, suggesting that young children use wealth cues to guide their evaluations of others during the preschool years. Additionally, responses on the wealth preference task did not differ by school SES, which suggests that the tendency to favor high-wealth individuals is not unique to children who have significant exposure to children from wealthier families. Nevertheless, in the absence of information about each participant’s family SES, it is difficult to draw conclusions about how children’s own backgrounds might affect the strength of their attitudes based on wealth cues. We address this question directly in Study 3.

Extending findings from previous studies with older children in the U.S. and South Africa [21,26], the younger participants in the present research were also more likely to match White (vs. Black) families with high-wealth stimuli. We can only speculate why White participants’ scores on the race/wealth matching task exceeded chance, while Black participants’ scores did not (but see [25], for a similar finding with older participants tested on implicit measures). One possibility is that Black children think White children have nicer possessions, but are also reluctant to pair members of their own group with less nice things. Such conflicting tendencies could lead to chance responding in the wealth/race matching task. Another possibility is that Black participants in Study 1 had different kinds or amounts of social contact with people from different racial and SES backgrounds. For example, relative to White participants, Black participants in Study 1 may have had more exposure to wealthy Black individuals or to poor White individuals. In future research, it would be useful to collect detailed information about children’s neighborhood racial and SES demographics, their personal experiences with people from different racial and SES backgrounds, and their exposure to television and other media.

## Study 2

The main aim for Study 2 was to conceptually replicate the findings from the wealth preference task in Study 1 with a new group of participants and stimuli, as well as a different dependent variable. As in Study 1, participants saw targets portrayed with objects that connoted greater or lesser wealth. Rather than indicating which person they liked, however, participants in Study 2 indicated which person they wanted to befriend. Asking about friendship decisions is a more direct measure of children’s social affiliation intentions.

Study 2 also tested whether young children simply prefer children presented with high-wealth cues, or whether they also use wealth cues to make inferences about other people’s attributes. We focused in particular on children’s wealth-based inferences about other people’s relative standing in two domains: academic competence and popularity. We chose these dimensions for two primary reasons. First, two well-documented beliefs on the part of adults are that poor people are incompetent and less well-liked by other members of society [29–31]. Second, familial SES is associated with both academic success and social prominence: Compared with children from low-SES families, children from middle- and high-SES families tend to perform better in school [32–33] and also tend to be a part of larger and more connected social networks [23].

On each of 9 trials in Study 2, participants learned about a different pair of target children who appeared to vary in wealth (as indicated by their material possessions). Across trials, participants answered questions about the targets’ other possessions (three trials), their relative competence (two trials), and their relative popularity (two trials); participants also indicated which target they would want as their own friend (two trials).

### Method

#### Participants

The participants in Study 2 were 48 4–6-year-old children (*N* = 24 males; *M* age = 66 months; range = 48–83 months) living in the Midwestern region of the U.S. The study was approved by the University of Wisconsin-Madison Social and Behavioral Sciences IRB [Protocol #2012–0764] and the Yale University IRB [Protocol #0807004095]). The legal guardians of all participants provided written informed consent, and children provided verbal assent. The majority of participants were White (94%). Caregivers were asked to provide information about family income and parental educational attainment on a questionnaire; two families did not provide income information and one family did not provide education information. Most participants (70%) came from families with incomes in excess of $75,000 a year, and 83% of participants had two parents with college degrees.

#### Materials

All trials included photographed faces of White children obtained through Internet searches. Faces within a trial were matched according to adult judgments of attractiveness, expression (all bore positive facial expressions), and age. Female participants saw photographs of young girls, while male participants saw photographs of young boys.

We used pictures to convey information about wealth, competence, and popularity to participants. Pictures of outfits represented targets’ wealth: “High-wealth targets” had items that were new and branded, while “low-wealth targets” possessed items that were used and generic. To imply competence, we showed pictures that appeared to have been colored by the targets. “High-competence” pictures featured objects that were colored the right color (e.g., a red apple) whereas low-competence pictures featured objects that had been colored the wrong color (e.g., an orange apple). Pictures of friend groups represented popularity. Each popularity stimulus pair included one picture with many faces of children (e.g., seven; “high popularity”) and one picture with fewer faces of children (e.g., two; “low popularity”). These stimuli were validated with a separate group of children (see S1 Text).

#### Procedure

Testing sessions took place in a lab room, with the participant seated at a table next to a White female experimenter. First, the experimenter showed participants a photograph of a classroom and said they would be learning about the children in that classroom. All participants then completed nine trials; each trial presented a unique pair of faces. Faces were presented on a computer screen against a white background. One face was located in the top left part of the screen, while the other was located in the top right part of the screen. For each trial, participants first learned a fact about each target (“fact learning”) and then answered just one question (“test”).

**Fig 2:**
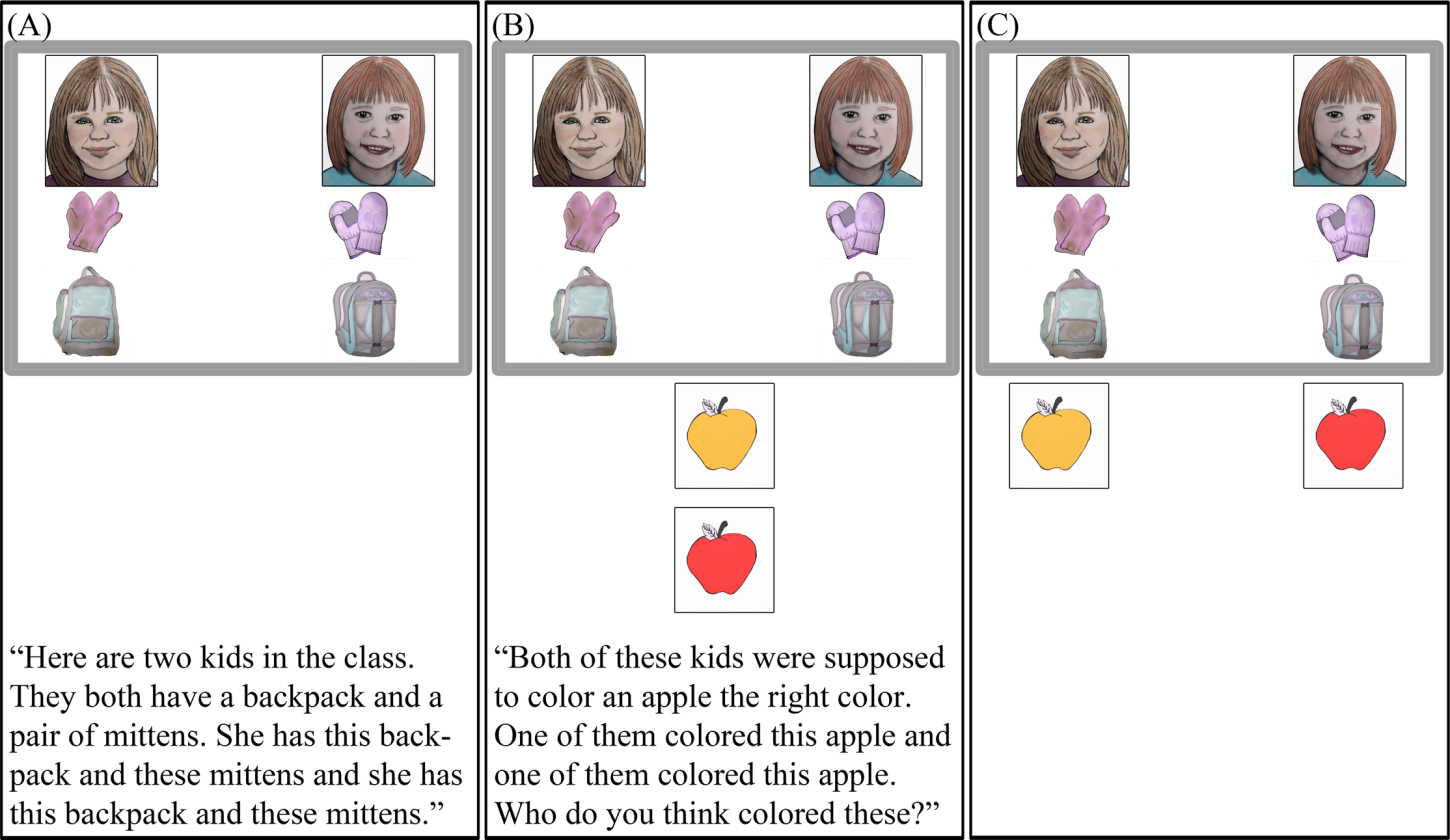
Example Trial from Study 2. (A) An example fact learning display; (B) An example wealth—> competence test display; (C) Here the high-competence stimulus is matched with the high-wealth target and the low-competence stimulus is matched with the low-wealth target. Because we do not have permission to publish the images that were used in the task, the figure shows an artist’s rendition of a display from the task. Participants in the study saw photographs of real objects and faces.

Fact learning: During the fact learning part of each trial, participants learned about two target children who differed in wealth. The experimenter never used social class labels to describe the targets or their possessions. Rather, participants saw that one target had an outfit that was new and branded (“high-wealth target”), while the other had an outfit that was used and generic (“low-wealth target”). At the beginning of each trial, the experimenter pointed to the two faces and said, “Here are two kids in the class. Both of these kids have [a sweatshirt and mittens].” Following this, pictures of new/branded items (e.g., a new, branded sweatshirt and mittens) appeared below one face (“high-wealth target”), while pictures of used/generic items (e.g., a used, generic sweatshirt and mittens) appeared below the other face (“low-wealth target”) on the computer screen. Starting on the left side of the screen, the experimenter pointed to the faces and items while saying, “S/he has [this sweatshirt and these mittens], and s/he has this [this sweatshirt and these mittens]” (Fig 2A). The experimenter then asked a test question.

Test: The experimenter presented test stimuli (printed on cardstock) at the participant’s midline on each trial (so that both cards were equidistant from each target; Fig 2B). Participants indicated their responses to the test questions by placing these cards beneath the faces of target children onscreen (Fig 2C).

The first three test questions for every participant concerned wealth (“wealth—>wealth inferences”). For the first pair of faces, participants were asked to make an inference about outfits owned by the targets (e.g., “Both of these kids also have a backpack and a pair of shoes. One of them has [this backpack and these shoes] and one of them has [this backpack and these shoes]. Who do you think has these?”). For the next two pairs of faces, participants made inferences about the targets’ other possessions. For one of the trials, participants matched pictures of high- vs. low- wealth (non-wearable) artifacts with targets (e.g., a new and branded doll + crayon set versus a used and generic doll + crayon set). For the other trial, participants matched pictures of high- vs. low-wealth family belongings with targets (e.g., a house that was large and well-maintained + a new car versus a house that was small and needed repairs + a car that was used).

The six remaining test questions were of three types: “wealth—>competence inferences” (two trials), “wealth—>popularity inferences” (two trials), and social preferences (two trials). For the “wealth—>competence inference” trials, participants heard that the target children were coloring in class that day, and were supposed to color a specific fruit or vegetable the right color, (e.g., “Both of these kids were supposed to color [an apple] the right color. One of the kids colored this and one of the kids colored this. Who do you think colored these?”). Participants were given cards—one with a correctly colored fruit/vegetable and one with an incorrectly colored fruit/vegetable—to indicate their responses. For the “wealth—>popularity inference” trials, participants were told that the target children did an activity with their friends, (e.g., “Both of these kids [went to the movies] with their friends. One of them went with [seven friends] and one of them went with [two friends]. Who do you think they went with?”). Participants were given cards—one with pictures of several children and one with pictures of fewer children—to indicate their responses. Popularity stimuli always showed faces of children who matched the participant’s gender. For the social preference trials, participants indicated which target they would prefer to have as their friend.

#### Design

Across trials within participants we counterbalanced the lateral positions of high- vs. low-wealth targets as well as the relative vertical positions of test stimuli (e.g., whether the low-competence stimulus card was closer to or farther away from the participant). Between participants we counterbalanced: the order in which particular face pairs appeared during the study; the pairings of particular targets with particular stimuli (i.e., sometimes a particular face was paired with a high-wealth outfit, and sometimes it was paired with a low-wealth outfit); and the order in which competence, popularity, and social preference test questions appeared during the session.

#### Scoring and data preparation

A score of “1” was assigned for matching the high-wealth, high-competence, or high-popularity stimulus card with the high-wealth target child on any given trial (which also meant matching a low-wealth, low-competence, or low-popularity stimulus card with the low-wealth target child). In contrast, a score of “0” was assigned for matching the low-wealth, low-competence, or low-popularity stimulus card with the high-wealth target child on any given trial (which also meant matching the high-wealth, high-competence, or high-popularity stimulus card with the low-wealth target child). Similarly, scores of “1” and “0” were assigned for selecting the high- or low-wealth target on social preference trials, respectively. All but one participant provided a response on every trial (one participant did not respond on either of the social preference trials).

### Results

Preliminary analyses revealed no effects of trial order, so this variable was not considered further. Because there were three different kinds of wealth—>wealth inference trials (outfits, non-wearable artifacts, and family belongings), each was analyzed separately using a sign test. There were two conceptually identical items for each of the other two kinds of inferences (wealth—>competence and wealth—>popularity) so participants’ responses on the two trials were combined for each inference type and analyzed with chi-square goodness of fit tests: The distribution of responses was compared to a chance distribution (by chance, a quarter of participants would achieve a score of 0, half of participants would achieve a score of 1, and a quarter of participants would achieve a score of 2).

**Fig 3:**
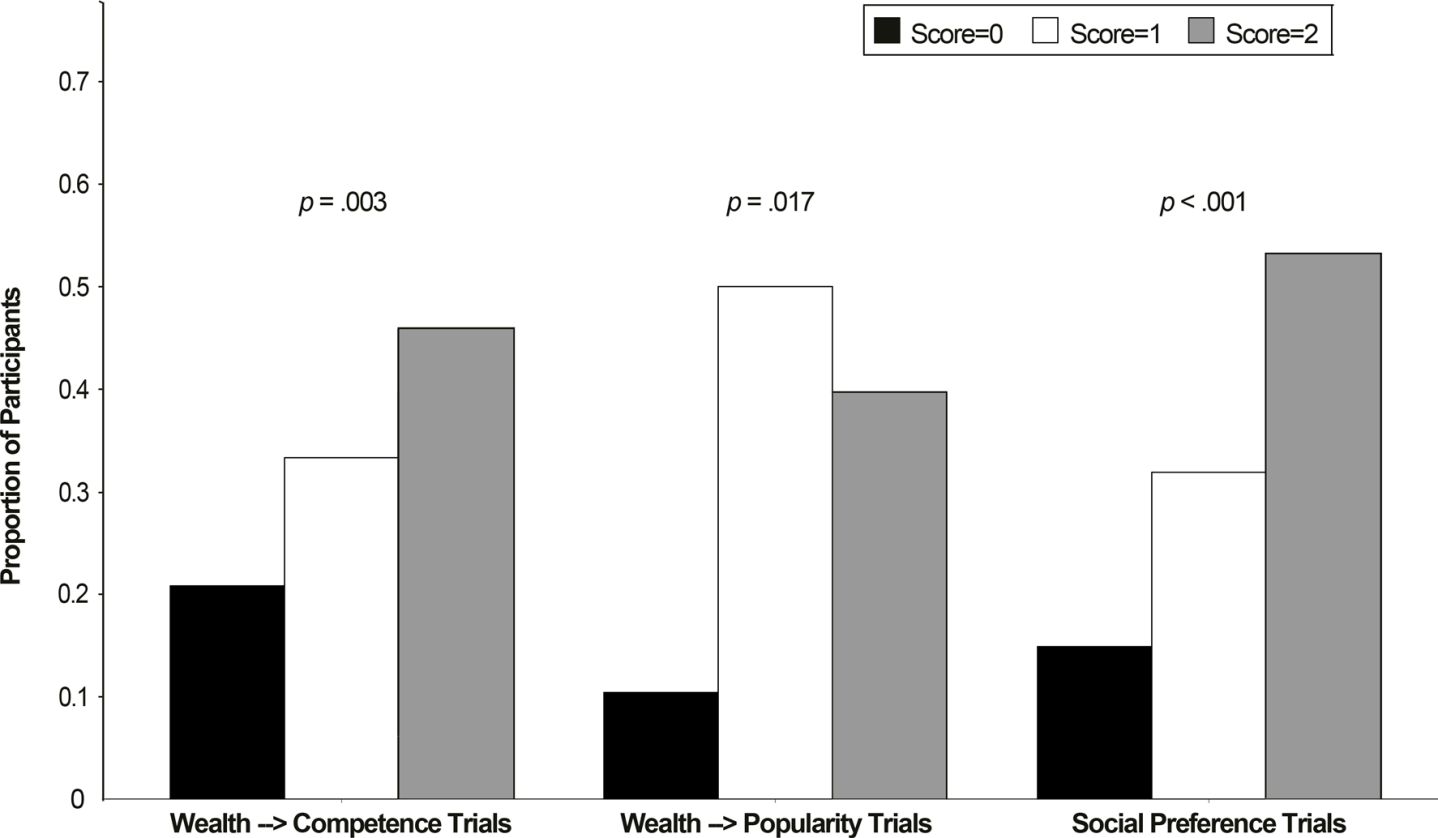
Responses of Participants in Study 2. The graph displays the proportion of participants in Study 2 who received scores of “0,” “1,” and “2” on different trial types. P-values note violations from the expected normal distribution; the normal distribution would be one quarter of participants with scores of “0,” half of participants with scores of “1,” and one quarter of participants with scores of “2.”

On each of the first three trials (wealth—>wealth inferences), the majority of participants indicated that the high-wealth target child was likely to own other high-wealth items (outfits question: 40 out of 48 children, *p* < .001; non-wearable artifacts question: 32 out of 48 children, *p* = .030; family belongings question: 33 out of 48 children, *p* = .014). Scores for both wealth—>competence inference trials and wealth—>popularity inference trials were different from the chance distribution, and skewed such that there were more high scores than would be expected by chance (*X*^2^(2, *N =* 48) = 11.33, *p* = .003, *w* = .49; *X*^2^(2, *N =* 48) = 8.17, *p* = 0.017, *w* = .41; respectively). Finally, participants tended to choose high-wealth targets as friends on social preference trials (*X*^2^(2, *N =* 47) = 19.94, *p* < .001, *w* = .65). Fig 3 displays participants’ performance in Study 2.

### Discussion

Participants indicated they would prefer to befriend children who were depicted with high-wealth possessions, replicating and extending the findings from Study 1. Children also indicated that someone with high-wealth possessions would be more likely than someone with low-wealth possessions to have other high-wealth possessions and family belongings, to complete a school assignment correctly, and to engage in activities with more social partners.

Although Studies 1 and 2 shed light on the early emergence of children’s consideration of cues to wealth, the methods did not allow us to address a number of important questions. First, because we could not collect information about families’ SES for participants in Study 1, and because participants in Study 2 came from families that were fairly high in SES, it is unclear whether to interpret the findings presented thus far as a preference for those who are wealthier, or as a preference for children who are most similar to participants themselves. Second, Studies 1 and 2 presented children with a forced-choice task where they had to choose between the high- and the low-wealth target on each trial. Although this procedure is common in studies of young children’s preferences, forced-choice presentations may overestimate or amplify children’s reliance on social differences when evaluating others. Third, Studies 1 and 2 presented pictures of material possessions to convey information about wealth to children, but children are likely exposed to other kinds of information about how wealthy or poor their peers are. For example, children may see that a classmate eats a school-provided breakfast each morning or may hear that a peer lives in a dangerous neighborhood. Do children use such information to evaluate others? Fourth, Studies 1 and 2 focused on very young children in order to probe whether such young children engage in wealth-based evaluations. The restricted age range (4–5 years in Study 1; 4–6 years in Study 2), however, made it difficult to test for any changes in children’s attitudes with development. To address these points, in Study 3 we included participants from diverse SES backgrounds; presented targets individually and asked participants to indicate their preferences on a scale; used short, varied descriptions to convey wealth information about targets; and tested children ranging in age from 5 to 9 years.

In addition to assessing children’s preferences in Study 3, we also included a measure designed to probe children’s behavior toward those who vary in wealth. In particular, children were asked to distribute resources (e.g., stickers) to targets. The literature offered competing hypotheses for children’s behavior in Study 3. On the one hand, young children are typically more generous toward people they like than they are toward people they dislike (e.g., friends vs. nonfriends; [34]). Yet, one recent study showed that liking and giving can dissociate: Li, Spitzer, and Olson [35] found that young children liked targets who had more possessions over those who had fewer (e.g., a girl with three jars of clay vs. a girl with one jar of clay), but tended to give more resources to those with fewer possessions (e.g., giving a stuffed animal to the child with less clay). Further, an older study [36] of children’s sharing behavior found that 4–6-year-old children from middle class homes shared more of their own resources with a target child described as coming from a “poor” family without money to buy good things than they did with a target child described as coming from a “rich” family with money to buy good things.

## Study 3

### Method

#### Participants

The participants were 65 children (29 boys, 36 girls) ranging in age from 5 to 9 years (*M* age = 89 months; range = 61 to 118 months). The study was approved by the University of Wisconsin-Madison Social and Behavioral Sciences IRB [Protocol #2012–0764] and the Yale University IRB [Protocol #0807004095]). The legal guardians of all participants provided written informed consent, and children provided verbal assent. As displayed in Table 1, participants came from a diverse range of household income brackets. All children from higher-income households and some of those from lower-income households where recruited from a database of families who had previously expressed interest in participating in child development research. Additional children from lower-income households were recruited through word-of-mouth and paper advertisements placed in neighborhoods and community centers serving lower-income families. Paralleling the demographics of the area where participants lived (a midsized city in the Midwestern region of the U.S.), most participants from higher-income households were White. Those from lower-income households came from more diverse racial and ethnic backgrounds (see Table 1 for more details). Three additional children participated in the study, but were not included in analyses because of equipment failure or experimenter error.

**Table 1:** Household Income and Race Information for Participants in Study 3.

Income Bracket	N	Race
Less than $25,000	21	13 Black; 3 Hispanic; 3 Black/White; 2 White
$25,000-$49,999	11	6 White; 2 Black; 2 Hispanic; 1 Black/White
$50,000-$74,999	3	3 White
$75,000-$99,999	9	7 White; 2 Asian
$100,000-$124,999	7	4 White; 2 Asian/White; 1 Hispanic
$125,000-$149,999	4	3 White; 1 Hispanic
$150,000-$174,999	5	5 White
$175,000-$199,999	2	2 White
$200,000 +	3	3 White

#### Materials

For each target, participants heard a short description of an experience common among children living in families with very limited finances or an experience common among children living in families with ample finances. The Appendix presents the full list of items and supporting references. Each item was pre-recorded by a woman and presented to children via computer speakers during the task. There were four descriptions (two “high-wealth” and two “low-wealth”) for each of the following six domains: food security, safety, housing, purchasing power, transportation, and free time. Descriptions were paired with photographed faces of Black, White, and Hispanic children during the task. Faces were similar in age, attractiveness, and expression. Female participants saw faces of girls only, and male participants saw faces of boys only.

Participants used a scale to indicate their preferences in the liking task. There were six points on the scale, each marked with a yellow face that differed in expression from very sad (to signify “really don’t like”) to very happy (to signify “really like”). In the giving task, participants could distribute up to five of each of the following kinds of toys: rubber ducks, jacks, erasers, dice, marbles, and rubber balls.

#### Procedure and design

Participants were tested in a quiet room in the lab or in a community center by a White female experimenter. The “liking task” was always presented before the “giving task.”

Liking task: The experimenter first introduced participants to the scale they would use to indicate their preferences during the task. She pointed to and labeled each point on the scale (1 = *really don’t like*; 2 = *don’t like*; 3 = *kind of don’t like*; 4 = *kind of like*; 5 = *like*; 6 = *really like*) and demonstrated how she would use the scale to indicate her preferences for different people (pointing to “6” for her “mom”, “5” for “a neighbor”, and “1” for “a mean person”). She then asked participants to name someone they really liked, someone they liked a little less than the person they really liked, and someone they really did not like, and to point to the corresponding faces on the scale. Next, the experimenter administered the test trials. On each trial, a rectangle on a computer screen raised to reveal a picture of target child. Participants then heard a pre-recorded description of the target. Once the recording ended, the target disappeared, the rating scale appeared, and the experimenter asked participants, “How much do you like this kid?”

Participants rated 12 different targets during the liking task (six presented with a high-wealth description and six presented with a low-wealth description). All participants rated two high-wealth and two low-wealth Black targets, two high- and two low-wealth Hispanic targets, and two high- and two low-wealth White targets. High- and low-wealth targets were interspersed throughout the task, as were faces of different races; the order of descriptions and faces varied across participants. Pairings of particular domains (e.g., transportation vs. food) to particular targets also varied across participants (e.g., some participants saw Hispanic child A with a low-wealth transportation description and some saw Hispanic child A with a high-wealth food description). Finally, some participants heard the 12 descriptions listed under “Set A” in the Appendix for the liking task, and some heard the 12 descriptions listed under “Set B” for the liking task.

Giving task: The experimenter first told participants that she had found some toys in her office earlier. She then told participants they would see and hear about some new kids, that they would be able to give as many or as few toys as they would like to each kid, and that any toys they did not give out would go back to the experimenter’s office. The experimenter then showed participants a picture of all of the toys they would have the opportunity to give away and explained a potential use for each toy (e.g., “Here are some jacks that kids can throw”). Participants then completed a practice trial featuring a cartoon dog and five toy bones. The experimenter used this trial to explain that participants should point to a red circle marked “all done” once they had finished giving out as many toys as they wanted on any given trial.

The beginning of each trial in the giving task was identical to the liking task. However, after the target disappeared from the screen, participants gave out toys rather than pointing to the scale. There were five toys of the same type (e.g., five rubber ducks) available to give out on each trial. Participants saw six different targets during the giving task (three targets presented with high-wealth descriptions: one Black, one Hispanic, and one White; three targets presented with low-wealth descriptions: one Black, one Hispanic, and one White). There were only six trials in the giving task (rather than 12) because piloting revealed that participants had difficulty completing more trials than that. The ordering and counterbalancing of trials was similar to that described for the liking task. Participants who heard the descriptions for Set A in the liking task heard a subset of the descriptions from Set B in the giving task. Participants who heard the descriptions for Set B in the liking task heard a subset of descriptions from Set A in the giving task.

### Results

Children’s race-based evaluations were not the focus of the present study; we only varied the race of faces so that most participants would see some faces in the task that matched their own background. Nevertheless, before proceeding with our main analyses, we first conducted two ANOVAs (one for the liking task, and one for the giving task) to test whether target race (Black, Hispanic, White) interacted with target wealth (low, high) in Study 3. There was not a significant interaction between target race and target wealth in either task (both ps > .396). Thus, target race was not considered in further analyses.

#### Liking task

Participants gave higher ratings to high-wealth targets than to low-wealth targets (*M* = 4.80, *SD* = .86 vs. *M* = 4.20, *SD* = 1.07, respectively; *t*(64) = 3.81, *p* < .001, *d*_*z*_ = .47). Table 2 displays the means for the different domain types (food, safety, housing, purchasing power, transportation, and free time). Paired-sample *t* tests (also displayed in Table 2) indicated that participants gave significantly higher ratings to high- than to low-wealth targets when descriptions referred to food, safety, housing, and free time. Ratings for high- and low-wealth targets were not significantly different when descriptions referred to purchasing power or transportation.

**Table 2:** Responses by Item Type in Study 3.

	*M* high-wealth (*SD*)	*M* low-wealth (*SD*)	Comparison
**Preference task**			
Food	4.94 (1.24)	4.37 (1.53)	*p* = .023, *d*_*z*_ = .29
Safety	5.14 (1.18)	3.62 (1.68)	*p* < .001, *d*_*z*_ = .76
Housing	4.78 (1.37)	4.37 (1.29)	*p* = .046, *d*_*z*_ = .25
Buying power	4.46 (1.55)	4.32 (1.53)	*p* = .616, *d*_*z*_ = .06
Transportation	4.57 (1.24)	4.29 (1.49)	*p* = .217, *d*_*z*_ = .16
Free time	4.91 (1.27)	4.25 (1.62)	*p* = .008, *d*_*z*_ = .34
**Giving task**			
Food	3.26 (1.18)	4.11 (1.29)	*p* = .009, *d*_*z*_ = .45
Safety	3.76 (1.13)	3.92 (1.32)	*p* = .588, *d*_*z*_ = .09
Housing	3.37 (1.24)	3.89 (1.19)	*p* = .110, *d*_*z*_ = .32
Buying power	3.11 (1.42)	3.85 (1.17)	*p* = .020, *d*_*z*_ = .48
Transportation	3.52 (1.34)	3.85 (1.10)	*p* = .232, *d*_*z*_ = .24
Free time	2.95 (1.58)	3.97 (1.26)	*p* = .004, *d*_*z*_ = .50

*Note*. Mean responses for “high-wealth” and “low-wealth” targets in Study 3. Standard deviations are noted in parentheses. The final column of the table presents p-values and effect sizes associated with paired samples t-tests comparing responses to “high-wealth” and “low-wealth” targets for each domain type within each task.

For analyses focused on participant age and participant family income, we created a difference score for each participant by subtracting average ratings given to low-wealth targets from average ratings given to high-wealth targets. There was not a significant correlation between participant age and the tendency to prefer high-wealth over low-wealth targets (*r =* .05, *p* = .705). Participant household income (binned into 9 brackets; see Table 1) and ratings for high- vs. low-wealth targets were also not significantly correlated (*r* = -.20, *p* = .102).

#### Giving task

Participants gave more resources to low-wealth than to high-wealth targets (*M* = 3.94, *SD* = .95 vs. *M =* 3.33, *SD* = 1.11; *t*(64) = 3.44, *p* = .001, *d*_*z*_ = .43). The average number of resources given to low-wealth (vs. high-wealth) targets was higher for all six domain types, but this difference was only significant when descriptions referred to food, purchasing power, and free time (see Table 2).

We created a difference score for each participant by subtracting the average number of resources given to high-wealth targets from the average number of resources given to low-wealth targets (positive scores therefore indicate giving more resources to low-wealth targets). Difference scores were not significantly correlated with participant age (*r =* .22, *p* = .085). However, there was a significant correlation between participant household income and performance in the giving task: Participants from wealthier families showed a stronger tendency to give more resources to low-wealth than to high-wealth targets (*r* = .27, *p* = .027).

### Discussion

The findings from Study 3 replicate and extend the results from the preference tasks in Studies 1 and 2. Children preferred high-wealth targets to low-wealth targets even though wealth information was conveyed with vignettes rather than with pictures, and even though targets were presented and evaluated individually (rather than in pairs). Further, the absence of a significant correlation between participants’ household income and responses on the liking task suggests that experiencing economic constraints in one’s own life does not attenuate the tendency to prefer those who are more economically advantaged. Taken together, the findings from Studies 1 and 3 cast doubt on the hypothesis that children’s preferences for high-wealth individuals can be explained by a preference for people or things that are more familiar or more similar to children themselves. In Study 1, there was no effect of school SES on children’s responses; those with more exposure to less wealthy individuals did not show a greater preference for low- over high-wealth targets. Further, in Study 3, there was not a significant relation between participants’ household income and responses on the liking task; if anything, children who came from less affluent families were more likely to prefer the wealthier targets (though the correlation was not significant).

In the giving task, participants tended to give more resources to low-wealth targets. This finding extends older research in which children tended to share more resources with those who were labeled as “poor” compared with those labeled as “rich” [36]. Further, the dissociation between children’s choices in the liking task (i.e., liking high-wealth targets) and their responses in the giving task (i.e., giving more to low-wealth targets) is consonant with research by Li and colleagues [35], in which children preferred those who had more resources but were more generous toward those with fewer resources. One possible explanation for such dissociations between liking and giving is that the underlying mechanisms are different: Preferences may be guided by automatic affective processes, while resource distribution decisions may be supported by conscious concerns about fairness (see [35] for discussion).

## General Discussion

### Summary

In the present work, children preferred people who were presented with high-wealth cues over those presented with low-wealth cues (Studies 1, 2, and 3), and paired high-wealth individuals with stimuli meant to symbolize academic competence and popularity (Study 2). White participants also paired White children with high-wealth cues and Black children with low-wealth cues. Moreover, children showed sensitivity to a wide range of cues associated with social class differences—from images of residences to pictures of personal effects to short descriptions of experiences (e.g., about food security and neighborhood safety).

The findings from the present research reveal children’s sensitivity to information that is ubiquitous in society, but often neglected by those interested in the development of social attitudes and stereotypes. Further, the results provide evidence that when children see individuals associated with the kinds of cues that connote social class (in society and in adults’ minds), they use such information to guide their preferences for, thoughts about, and actions toward, other people. There are, however, several remaining questions and important directions for future research on children’s consideration of social class information.

### Limitations and Future Directions

An important remaining question concerns why children preferred individuals presented with high-wealth cues in the present research. One possibility is that children understood the high-wealth and low-wealth cues as socioeconomic indicators and thus used socioeconomic information per se to guide their responses in each task. For example, upon seeing a child with a new, branded backpack, participants may have thought, “This kid has a lot of money” or “This kid’s dad probably has a really good job.” Another possibility, however, is that children simply liked (what we call) the high-wealth objects and stories more than they liked (what we call) the low-wealth objects and stories. Positive feelings toward (what we call) the high-wealth stimuli may have led them to: (a) like the individuals associated with such stimuli; and (b) match such stimuli with other positively valenced stimuli (e.g., White faces for White children in Study 1, and the “high competence” and “high popularity” stimuli in Study 2).

The present studies cannot distinguish between a richer (socioeconomic interpretation) and leaner (positive associations) explanation of children’s responses. However, we do note the following: (1) Children in the stimulus validation task for Study 2 did point to the high-wealth (rather than the low-wealth) stimuli when asked which things belonged to children whose parents had more money and could buy them whatever they wanted. This suggests that they may have viewed the stimuli as conveying socioeconomic information. That said, we cannot know what participants in Study 2 were thinking, and participants in the stimulus validation task for Study 2 may have answered our questions using a simpler heuristic (e.g., match the nicer item with the phrase “whatever they want”). (2) Children in Study 3 gave more material resources to children who were introduced with low-wealth information. This behavior suggests that participants may have understood that such children were likely to have fewer resources. Further, children’s giving to the low-wealth targets in Study 3 could be thought of as matching positive stimuli (i.e., fun objects) with less positive stimuli (i.e., low-wealth facts). Nevertheless, the leaner interpretation of our findings cannot be eliminated. Further, it may be the case that social class biases in the real world emerge from a tendency to disfavor items and experiences that are commonly associated with lower socioeconomic status (e.g., through affective, associative processes [35]): Just as merely appearing next to a morally good person or experiencing a lucky yet uncontrollable event (e.g., winning the lottery) can lead to more positive evaluations of a spatially contemporaneous or affected person [37–39], merely appearing in the context of higher-wealth items may lead to more positive evaluations of wealthier people.

Further research is necessary in order to shed light on the merits of different explanations for children’s responses. For example, to test whether affective associations play a role in guiding children’s preferences, future studies might investigate whether children also like people who are merely associated with high-wealth stimuli, but who are not, in fact, wealthy (e.g., people looking at books with pictures of nice houses). Future research might also present stimuli that connote high wealth, but that are not in fact desirable to young children (e.g., a Gucci purse).

Another question for future research concerns the range of cues that may guide children’s evaluations of those who are higher in status. In the present research, children showed social preferences based on information about the nature of other people’s material possessions (Studies 1 and 2), as well as information about daily advantages and hardships associated with having more or less money (Study 3). However, infants and children are sensitive to other manifestations of social status, including dominance, power, and occupational prestige (e.g., [24,40–42]. Do other status cues influence children’s social preferences?

### Conclusions

As early as the preschool years (and continuing into adulthood), people tend to like those who appear to be advantaged more than those who appear to be disadvantaged. Thus, in addition to well-documented problems in their physical environment (e.g., increased pollution, less access to healthy food; [23,43–44]), children from poor families likely experience challenges in their social environments (e.g., when making friends at school). A more thorough understanding of the sources and nature of wealth-based evaluations may eventually shed light on how to improve the social experiences of children from such financially disadvantaged families.

Beyond contributing to our understanding of the range of factors that children use to evaluate individuals, research on children’s consideration of those who vary in status can shed light on mechanisms underlying children’s evaluations of social groups. A growing body of research provides evidence that children represent links between racial groups and SES (e.g., [14,21,24–25]) and show preferences in favor of groups that are higher in SES (e.g., liking Whites over Blacks in South Africa and the United States; [45–46]. Additionally, in a recent study, preschool-age children who learned that members of one novel group (e.g., “the Oranges”) were wealthy while members of another novel group (e.g., “the Greens”) were poor subsequently preferred new members of the wealthier group in a test phase [20]. Thus, children’s status perceptions and evaluations appear to play a role in guiding their attitudes toward social groups from an early age.

## Appendix

The following items describe common experiences of children from families that are higher vs. lower in socioeconomic status (see [22,23,47–52]).

### Food Items

Set A:

High: This kid’s family always has enough food; she/he eats breakfast at home before school.

Low: This kid’s family only has a little food; she/he eats breakfast when she/he gets to school.

Set B:

High: This kid’s family always has enough food; she/he can pay attention at school because she/he has a full stomach.

Low: This kid’s family only has a little food; she/he has trouble paying attention at school because she/he is hungry.

### Safety Items

Set A:

High: This kid’s neighborhood is safe; in the summer, there are people walking with their kids and dogs at night.

Low: This kid’s neighborhood is dangerous; in the summer, there are not many families walking around at night.

Set B:

High: This kid’s neighborhood is safe; she/he can always fall asleep at night because it is quiet and there is not a lot of traffic.

Low: This kid’s neighborhood is dangerous; she/he sometimes has trouble falling asleep at night because it’s noisy and there is a lot of traffic.

### Housing Items

Set A:

High: This kid lives in a big house; she/he and her/his two sisters/brothers all have their own rooms.

Low: This kid lives in a small apartment; she/he and her/his two sisters/brothers share a room.

Set B:

High: This kid lives in a big house; she/he and her/his two brothers have a very big playroom that they can play in.

Low: This kid lives in a small apartment; she/he and her/his two brothers have to play in the room they share.

### Purchasing Power Items

Set A:

High: This kid’s family has a lot of money; when her/his shoes were too small she/he could get new ones at the store right away.

Low: This kid’s family only has a little money; when her/his shoes were too small she/he had to wait a long time to get shoes that fit.

Set B:

High: This kid’s family has a lot of money; when her/his shirt is too small she/he can go to the mall to pick out a new one right away.

Low: This kid’s family only has a little money; when her/his shirt is too small she/he has to wait a long time to get a shirt that fits her/him.

### Transportation Items

Set A:

High: This kid’s mom has her own car; she/he and her/his mom get to drive in her car to the grocery store.

Low: This kid’s mom doesn’t have a car; she/he and her/his mom have to take two buses to the grocery store.

Set B:

High: This kid’s mom has her own car; her/his mom drives her/him to school in the morning.

Low: This kid’s mom doesn’t have a car; she/he takes two city buses with his mom to school.

### Free Time Items

Set A:

High: This kid’s parents have jobs that pay them a lot of money; she/he and her/his family went to Disney World in Florida for vacation.

Low: This kid’s parents have jobs that only pay them a little money; she/he and her/his family stayed home and watched T.V. for vacation.

Set B:

High: This kid’s parents have jobs that pay them a lot of money; she/he and her/his family went on a Disney Cruise to the beach for vacation.

Low: This kid’s parents have jobs that only pay them a little money; she/he and her/his family stayed home and watched movies for vacation.

## Supporting Information

S1 Data Data File for Study 1. (XLSX)

S2 Data Data File for Study 2. (XLSX)

S3 Data Data File for Study 3. (XLSX)

S4 Data Data File for Stimulus Validation (Study 2). (XLSX)

S1 Permission Permission to publish the images in Fig 1 and Fig 2. (DOCX)

S1 Text Description of Stimulus Validation (Study 2). (DOCX)
