# Supplementary material for: Children Use Wealth Cues to Evaluate Others
Source: PLoS One. 2016 Mar 2;11(3):e0149360. doi: 10.1371/journal.pone.0149360 (PMC4774995; doi:10.1371/journal.pone.0149360)
Supplement: S1 Permission — (DOCX) [file pone.0149360.s005.docx]

---------- Forwarded message ----------

From: **Rachel King**

Date: Mon, Nov 30, 2015 at 5:24 PM

Subject: Re: Request permission for drawings

To: Libbie Brey <[elizabeth.brey@gmail.com](mailto:elizabeth.brey@gmail.com)>

Dear Elizabeth,

You have my permission to use my drawings, "Figure 1" and "Figure 2" for the open-access journal PLOS ONE under the Creative Commons Attribution License (CCAL) CC BY 3.0.  I understand that this license allows unrestricted use and distribution of these drawings, even commercially, by third parties.

Please let me know if there is anything else I need to do to give you permission to use my drawings.

Best,

Rachel King

On Mon, Nov 30, 2015 at 4:43 PM, Libbie Brey

Dear Rachel,

I request permission for the open-access journal PLOS ONE to publish your drawings "Figure 1" and "Figure 2" under the Creative Commons Attribution License (CCAL) CC BY 3.0 (<http://creativecommons.org/licenses/by/3.0/us/>). Please be aware that this license allows unrestricted use and distribution, even commercially, by third parties. Please reply and provide explicit written permission to publish these two images under a CC BY license.

Thank you,

Elizabeth

--

Libbie Brey

Graduate Student in Psychology

University of Wisconsin - Madison

1202 West Johnson Street Rm. 177

Madison, WI 53706

[elbrey@wisc.edu](mailto:elbrey@wisc.edu)

---------- Forwarded message ----------

From: **Kara Weisman**

Date: Mon, Nov 23, 2015 at 10:25 PM

Subject: Re: Request permission for drawings

To: Libbie Brey <[elizabeth.brey@gmail.com](mailto:elizabeth.brey@gmail.com)>

Dear Elizabeth,

I am writing to provide you permission to publish these two images under a Creative Commons Attribution License CC BY 3.0.  I created both of these drawings and I give you permission for PLOS ONE to publish them.  Please let me know if you need any other information from me.

Best wishes,

Kara

On Mon, Nov 23, 2015 at 5:00 PM, Libbie Brey

Dear Kara,

I request permission for the open-access journal PLOS ONE to publish your drawings "High SES house" and Low SES house" under the Creative Commons Attribution License (CCAL) CC BY 3.0 (<http://creativecommons.org/licenses/by/3.0/us/>). Please be aware that this license allows unrestricted use and distribution, even commercially, by third parties. Please reply and provide explicit written permission to publish these two images under a CC BY license.

Thank you,

Elizabeth



--

Elizabeth Brey

Graduate Student in Psychology

University of Wisconsin - Madison

1202 West Johnson Street Rm. 177

Madison, WI 53706

[elbrey@wisc.edu](mailto:elbrey@wisc.edu)
