# Supplementary material for: Children Use Wealth Cues to Evaluate Others
Source: PLoS One. 2016 Mar 2;11(3):e0149360. doi: 10.1371/journal.pone.0149360 (PMC4774995; doi:10.1371/journal.pone.0149360)
Supplement: S1 Text — (DOCX) [file pone.0149360.s006.docx]

**Stimulus Validation (for Study 2)**

To assess whether young children would interpret the pictures as intended in Study 2, we paired the wealth, competence, and popularity stimuli (described in the main text) with faces of unfamiliar target children and asked a group of children (*N* = 48; 26 males; *M* age = 5.51 years; range = 4.10–6.92 years; 94% White; all tested in the Midwestern region of the U.S.) a series of questions. None of these children participated in Studies 1, 2, or 3.

For wealth stimulus pairs, participants were asked, “Which one of these kids’ parents do you think has more money and can buy him/her whatever he/she wants?” For competence stimulus pairs, participants were asked, “Which one of these kids do you think is smarter and knows a lot?” For popularity stimulus pairs, participants were asked, “Which one of these kids do you think is more popular and well liked?”

Each participant saw four pairs of each stimulus type (competence, wealth, and popularity) and a score was calculated for each stimulus type by combining across the four trials. Total scores for each stimulus type ranged from “0” to “4.” Participants rarely said “I don’t know” (N = 3) and a score of 0.5 was assigned on those trials.

Participants pointed to targets with high-wealth outfits on wealth trials, to targets with correctly-colored produce on competence trials, and to targets with larger friend groups on popularity trials (chance = 2; *M* _Wealth Pairs_ = 3.56, *SD* = .76, *t*(47) = 14.20, *p* < .001, *d* = 2.05; *M* _Competence Pairs_ = 3.89, *SD* = .31, *t*(47) = 41.66, *p* < .001, *d* = 6.10; *M* _Popularity Pairs_ = 3.63, *SD* = .89, *t*(47) = 12.65, *p* < .001, *d* = 1.83).
